# Monitoring of Pathogens Carried by Imported Flies and Cockroaches at Shenzhen Ports

**DOI:** 10.3390/tropicalmed10020057

**Published:** 2025-02-17

**Authors:** Siqi Zhang, Chunzhong Zhao, Guoping Liu, Liwei Guo, Ran Zhang, Junyu Yan, Jianan He, Cheng Guo

**Affiliations:** 1 College of Animal Science and Technology, Yangtze University, Jingzhou 434025, China; 13080666539@163.com (S.Z.); guoping.liu@yangtzeu.edu.cn (G.L.); guolw@yangtzeu.edu.cn (L.G.); junyuyan999@163.com (J.Y.); 2 Shenzhen Customs District Port Outpatient Clinics, Shenzhen International Travel Health Care Center, Shenzhen 518000, China; forrestszx@icloud.com (C.Z.); zhangrank@163.com (R.Z.); 3 School of Public Health, Sun Yat-sen University, Guangzhou 510080, China

**Keywords:** vectors, metagenomic sequencing, enteric pathogens, respiratory pathogens, mechanical carriage

## Abstract

This study tested the efficacy of xenomonitoring using contaminated flies and cockroaches at ports in Shenzhen by analysing sample data from imported flies and cockroaches from October 2023 to April 2024 to identify the pathogens they carried. Among all the samples of flies and cockroaches collected, *Musca domestica vicina* and *Blattella germanica* accounted for the highest proportion, 27.59% and 66.47%, respectively. Their positive rates for carrying *Staphylococcus aureus* were also the most significant, reaching 4.35% and 6.47%, respectively. The imported flies and cockroaches mainly came from Asia, with the highest proportion coming from Hong Kong, at 97.71% and 92.11%, respectively. Metagenomic sequencing indicated that the pathogens carried by the flies and cockroaches from different regions of Asia were generally similar but showed some differences. Flies from Southeast Asia, East Asia, South Asia, and West Asia and cockroaches from Southeast Asia, East Asia, and West Asia harboured unique opportunistic pathogens capable of causing gastrointestinal and respiratory infections in humans. Specifically, flies carried pathogens such as *Campylobacter jejuni*, *Bacillus anthracis*, *Bacteroides fragilis*, and *Bordetella bronchiseptica*, while cockroaches carried *B. fragilis*, *Clostridium tetani*, and *Bacillus cereus*. Our findings provide data support for future risk assessments of pathogens carried by imported vectors.

## 1. Introduction

Worldwide, vector-borne diseases (VBDs) have greatly affected humans, as over 80% of the global population resides in regions vulnerable to at least one VBD. VBDs are infections caused by pathogens transmitted by arthropods [1,2]. According to the WHO report, 70% of the world’s infectious diseases are insect-borne diseases, causing hundreds of millions of infections and more than 2 million deaths every year, resulting in a global disease burden and economic losses of hundreds of billions of dollars. In particular, between 1.4 million and 4.3 million people worldwide are infected with cholera each year, while between 280,000 and 1.42 million people die from cholera infections each year. Flies and cockroaches are common port vectors capable of transmitting various pathogens, including bacteria, viruses, and parasites, and are mechanical carriers of multiple enteric pathogens [3,4]. Some Chinese cities, such as Shenzhen, contain major ports that receive ships from around the world. Shenzhen itself has 16 ports that are active in foreign trade and international exchanges. Hence, VBDs have become a major public health threat in China [5].

Various pathogens evolve frequently through mutation and recombination [6]. Regular nucleic acid-based monitoring and testing of such viruses is, therefore, particularly important to address emerging viruses and changing global viral diversity. A virus-monitoring system called “xenommonitoring” [7] has emerged. In the past, pathogen surveillance systems were costly, it was difficult to sample enough different host species in different spaces and times, and vectors were introduced to new areas through various forms of transportation, which accelerated the spread of outbreaks along with vector activity and potentially disrupted local ecosystems. The system uses flies and cockroaches contaminated by surface mechanical transmission or active ingests of spreading viruses as external surveillance media [8,9], and through the combination of metagenomics, it is not only relatively cheap but also provides a deeper understanding of the virus status in the monitored environment and the source of imported insects, thus providing valuable epidemiological information.

This study aimed to understand the viruses entering China via Shenzhen using xenomonitoring to analyse the pathogens in imported insect vectors. The whole early warning evaluation process is shown in Figure S1. The biological importance of detecting vector samples was investigated, and the data were converted into actionable insights for public health. The goal is to strengthen the control of flies and cockroaches at ports, detect and intercept foreign species in time, and provide early warning of potential sources of imported pathogens so that timely measures can be taken to prevent and control them, thereby reducing the risk of invasion and spread of foreign infectious diseases. Strengthening the health and quarantine work on entry transport vehicles and the control of vectors at ports can detect and deal with disease-carrying vectors in time, effectively prevent the spread of diseases, protect people’s lives and health, and prevent the introduction and spread of diseases through ports, which is of great significance for safeguarding national health and economic production.

## 2. Materials and Methods

### 2.1. Sample Collection, Composition Ratio, and Statistical Analysis of Origins

#### 2.1.1. Sample Collection

Samples of flies and cockroaches intercepted at 13 ports in Shenzhen, China, between October 2023 and April 2024 were obtained. According to the Regulations on Medical Vector Monitoring at Border Ports and Entry-Exit Vehicles, the monitoring methods for flies and cockroaches involved using net capture techniques to catch flies and cockroaches on inbound vehicles, such as ships and aeroplanes, as well as in cargo containers. The captured samples were placed in sterilised 5 mL EP tubes and stored in a freezer at −80 °C.

#### 2.1.2. Composition Ratio and Origin Distribution

All captured vector samples were classified and statistically analysed by species, type, quantity, and place of origin. The data were organised into an Excel spreadsheet and subjected to graded statistical analysis through the ArcGIS 10.2 software from ESRI, Redlands, CA, USA.

### 2.2. Main Reagents and Instruments

The following kits were used: the polymerase chain reaction (qPCR) detection kits for *Vibrio cholerae*, *Salmonella*, *Shigella*, *Escherichia coli* O157, and *Staphylococcus aureus* (Daan Gene Co., Ltd., Guangzhou, China); the HiPure Insect DNA Kit (a column-based nucleic acid extraction kit) (Magen, Guangzhou, China); the MGIEasy Enzymatic DNA Library Prep Kit; the DNBSEQ OneStep DNB Make Reagent Kit; the MGISEQ-200RS High-Throughput Sequencing Reagent Kit (BGI Genomics Shenzhen Technology Co., Ltd., Shenzhen, China); and the S3 fragment cartridge kit (Houze Bio-technology Co., Ltd., Shenzhen, China).

The following instruments were used: a MagNA Lyser homogeniser (Roche, Risch-Rotkreuz, Switzerland); a NanoDrop 2000 ultra-micro spectrophotometer (Thermo, Waltham, MA, USA); a Qubit 4 fluorometer (Thermo USA, MA); an ABI 7500 Real-Time Quantitative PCR Instrument (Thermo, USA, MA); the MGISP-100RS Automated Library Preparation System (MGI Tech Co., Ltd., Shenzhen, China); an MGISEQ-200RS sequencer (MGI Tech Co., Ltd., Shenzhen, China); and a Qsep100 fully automated nucleic acid protein analysis system (BiOptic Inc., Taiwan, China).

### 2.3. DNA Extraction

Samples were thawed at 25 °C for 10 min, after which 20 mg of each sample was placed in a 2 mL EP tube. Zirconium oxide and 1 mL of physiological saline were added, and the mixture was homogenised for 1 min to generate a tissue homogenate. Genomic DNA was extracted according to the manufacturer’s instructions for the HiPure Insect DNA Kit.

### 2.4. Quantitative Real-Time PCR Detection of Pathogens

The nucleic acids of *V. cholerae*, *Salmonella*, *Shigella*, *E. coli* O157, and *S. aureus* in the samples were detected through the PCR-fluorescent probe method as follows. The reaction system had a total quantity of 460 nM, which included 250 nM of the reaction premix, 200 nM of the enzyme mix, and 10 nM of the sample nucleic acid. The reaction programme involved reverse transcription (50 °C for 2 min), inactivation of reverse transcriptase and initial denaturation (95 °C for 15 min), denaturation (94 °C for 15 s), annealing and fluorescence acquisition (55 °C for 45 s), extension (72 °C for 15 s), and amplification for a total of 40 cycles. The detection channels for *V. cholerae*, *Salmonella*, *E. coli* O157, and *S. aureus* were FAM, and the detection channel for *Shigella* was VIC.

### 2.5. Nucleic Acid Quality Control

The integrity of the DNA from 106 samples (53 fly samples and 53 cockroach samples) was assessed through a fully automated nucleic acid and protein analysis system for quality control. Samples with a DQN value of >6 were considered to be of acceptable quality.

### 2.6. Library Construction and Metagenomic Sequencing

According to the manufacturer’s instructions, the MGIEasy Enzymatic DNA Library Prep Kit was used to construct libraries with 15 μg of high-quality DNA. The DNA was randomly fragmented, and fragments between 200 and 400 bp were selected and purified using AMPure XP beads [10]. The selected DNA fragments were then ligated with PE indices, and the ligated DNA was purified. The purified ligated DNA was circularised, followed by rolling circle amplification to generate DNA nanoballs, which were then sequenced on the MGISEQ-200 platform through a PE100 bp strategy.

### 2.7. Raw Data Processing and Bioinformatics Analysis

The type of vector species was entered into Genomes–NCBI Datasets accessed on 18 November 2024 (https://www.ncbi.nlm.nih.gov/datasets/genomes/) [11] to identify the corresponding species sequence, and one of them was selected as the host sequence. First, the low-quality reads (with a low base quality and a high percentage of N bases) were filtered, and adapters were cut using SOAPnuke (version 2.1.7). Then, the host reads were identified and filtered by aligning filtered reads to the host reference genome using snap-aligner (version 2.0.1). SortMeRNA (version 2.0) was used to remove rRNA reads from the RNA sample. After filtering out host (and rRNA) reads, taxonomic classification was performed on the clean reads using Kraken2 (version 2.1.2) and estimated abundance with Bracken (version 2.5). After obtaining fastQ data, low-quality unqualified reads were filtered, and high-quality reads were retained for genome assembly.

The factors considered in the filtration process included the deletion of low-average-base-mass reads and a high proportion of N bases. Viral samples may have had repeated reads. The pipeline allowed us to maintain low virtual memory consumption and calculate the time when running genome assembly. Finally, a re-sequencing analysis was performed using the reference genome provided. The output results included mutation call files (SNPs, Indel in VCF format) and consensus genome sequences. The assembled sequences were analysed using MGAP. The assembled sequences were based on the list of human-borne pathogenic microorganisms issued by the National Health Commission of the People’s Republic of China [12]. The data were collated using Python 3.13.2, and the data were visualised using TBtools v2.056, MEGA 7.0., and Excel.

## 3. Results

### 3.1. Analysis of Composition Ratio and Origin Distribution of Imported Vectors

**Figure 1:**
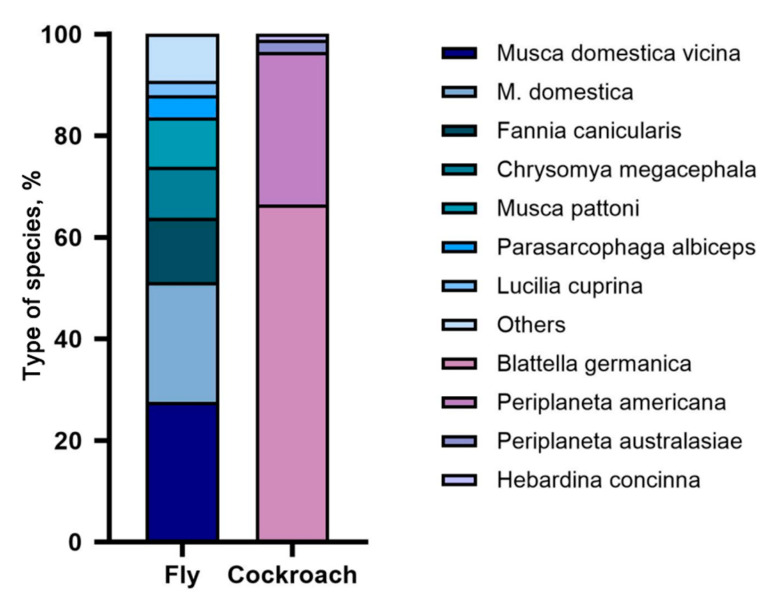
Overview of imported vectors at Shenzhen ports.

A statistical analysis was conducted on 518 insects intercepted during monitoring at Shenzhen ports, comprising 348 flies and 170 cockroaches (Figure 1). The fly samples included 6 families, 9 genera, and 14 species. Among these, *Musca domestica vicina* had the highest proportion at 28% (96/348), followed by *M. domestica* at 23% (82/348), *Fannia canicularis* at 13% (44/348), *Chrysomya megacephala* at 10% (35/348), and *Musca pattoni* at 10% (34/348). Other species accounted for smaller proportions, including *Parasarcophaga albiceps* at 4% (15/348), *Lucilia cuprina* at 3% (10/348), *Fannia leucosticte* at 2% (8/348), *Anthomyia illocata* at 2% (8/348), *Neomyia timorensis* at 2% (6/348), *Lucilia sericata* at 2% (6/348), *Sarcophagidae* at 0.57% (2/348), *Musca sorbens* at 0.29% (1/348), and *Syrphidae* at 0.29% (1/348). The cockroach samples included two families, three genera, and four species. Among these, *Blattella germanica* had the highest proportion at 67% (113/170), followed by *Periplaneta americana* at 30% (51/170). Other species accounted for smaller proportions, including *Periplaneta australasiae* at 2% (4/170) and *Hebardina concinna* at 1% (2/170).

**Figure 2:**
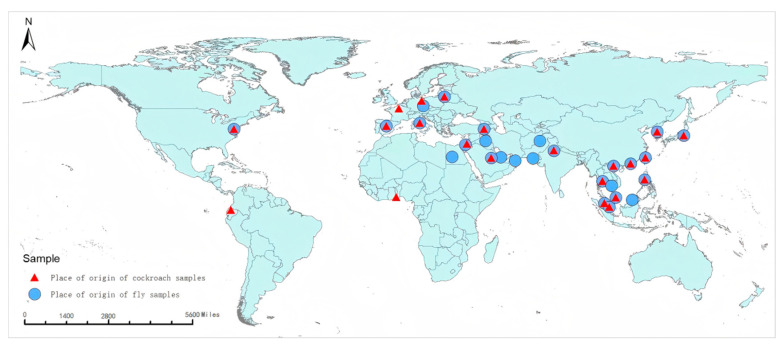
Origins and distribution of fly species and cockroach species.

Based on data from the places of origin of the samples, GIS maps downloaded from the Department of Natural Resources Standard Mapping Service website were divided into five levels, represented by circles and triangles of different sizes at marked latitudes and longitudes. Based on administrative geographical divisions, the samples’ origins were categorised into five continents (Asia, Europe, Africa, South America, and North America) to express the proportion of samples from each country and geographical division. Most flies and cockroaches originated from Asia, with proportions of 97.71% and 92.11%, respectively. Specifically, within Asia, Hong Kong had the highest proportion, with 38.51% for flies and 52.73% for cockroaches. For the flies, Cambodia, the Czech Republic, Qatar, the United States, South Africa, and Thailand had the lowest proportions, each accounting for 0.29%, with only 1 fly captured from each location. For cockroaches, Togo, Saudi Arabia, Italy, Germany, Israel, India, and Ecuador had the lowest proportions, each accounting for 0.61%, with only 1 cockroach captured from each location (Figure 2).

### 3.2. Pathogen Detection Status

The flies had the highest positive rate for *S. aureus*, at 4.35% (15/348). Among them, the positive rate was most notable in *L. cuprina* at 10% (1/10). Furthermore, *S. aureus* was detected in *P. albiceps*, *M. domestica*, *Chrysomya megacephala*, *M. domestica vicina*, and *M. pattoni*, with positive rates of 6.67% (1/15), 6.25% (6/96), 5.71% (2/35), 4.17% (4/96), and 2.94% (1/34), respectively.

The positive rate for *E. coli* O157:H7 was 1.15% (5/435), and it was detected only in *M. domestica vicina* and *M. domestica*, both with a positive rate of 2.08% (2/96 and 2/82, respectively). *V. cholerae* had the lowest detection rate at 0.57% (2/348), found only in *P. albiceps* (6.67%, 1/15) and *F. canicularis* (2.27%, 1/44). Neither *Salmonella* nor *Shigella* were detected.

Among the cockroaches, the highest positive rate detected was for *S. aureus*, at 6.47% (11/170). *B. germanica* showed the most notable positive rate at 8.85% (10/113), and *Periplaneta americana* had a positive rate of 1.96% (1/51). Additionally, *V. cholerae* and *Shigella* were detected in *B. germanica*, each with a positive rate of 0.88% (1/113). Neither *Salmonella* nor *E. coli* O157:H7 was detected (Table 1 and Table 2).

### 3.3. Differences in the Composition of Pathogens Carried by Imported Flies and Cockroaches from Different Regions

**Figure 3:**
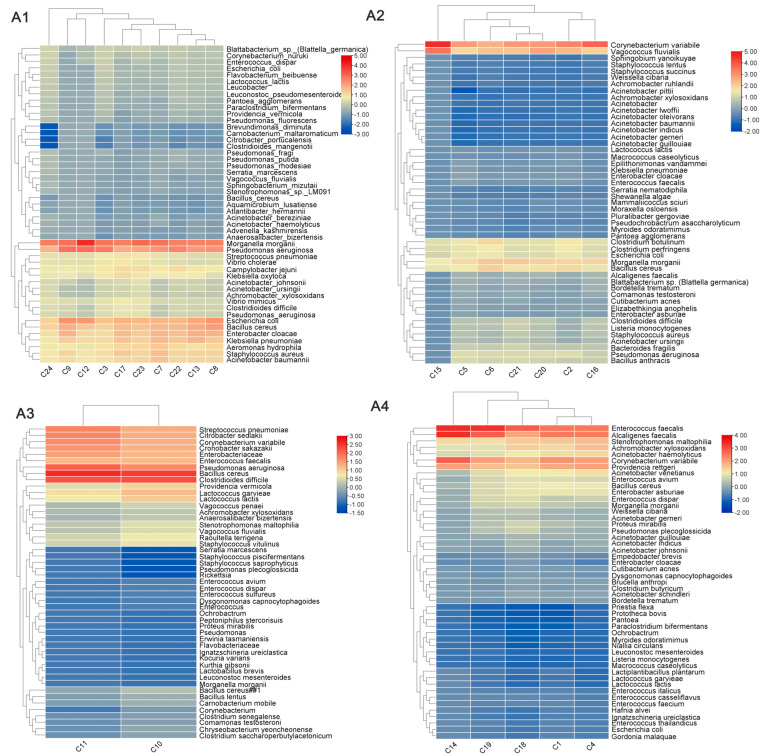

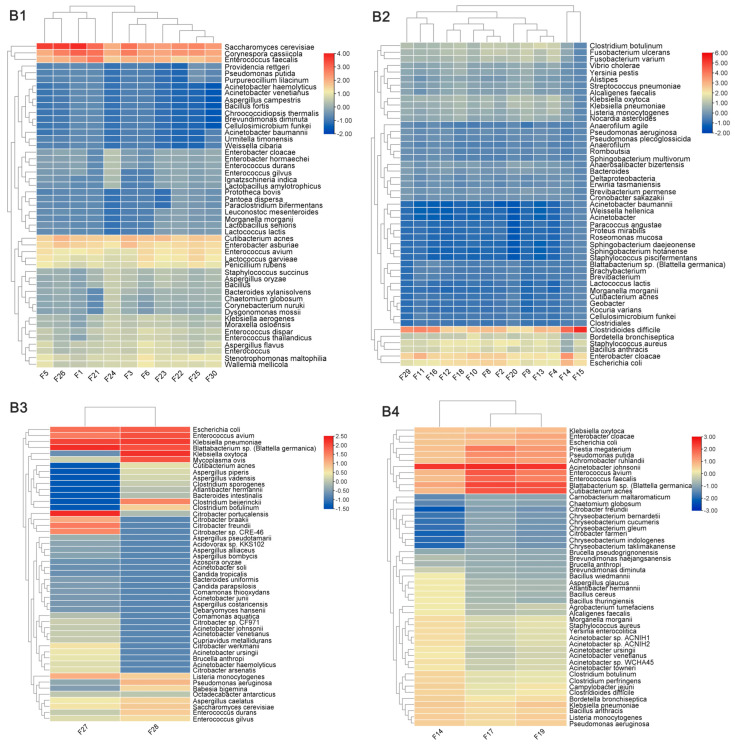
Heatmap analysis of pathogen levels carried by flies and cockroaches from different Asian regions. (**A**) Flies and (**B**) cockroaches; regions 1–4 correspond to Southeast Asia, East Asia, South Asia, and West Asia, respectively. The names of 50 bacterial species are listed in the right column, and the names of different samples are at the bottom. The colour indicates the base−10 logarithm of the sequence read count, ranging from blue (a low read count) to red (a high read count).

In total, 54 samples (24 fly samples and 30 cockroach samples) had a DQN of >6 and were deemed acceptable samples suitable for subsequent sequencing analysis. After quality control and removal of host DNA contamination, an average of 78 M reads were measured per sample for downstream analysis (Figure S2). All samples that passed quality control were categorised by region, encompassing one continent (Asia) and four regions (East Asia, West Asia, South Asia, and Southeast Asia). The analysis was conducted separately for flies and cockroaches across these four regions. “Reads per million” (RPM, per million mapped reads) was used to measure the abundance of infection in vivo, with microorganisms having an RPM of >1 considered present to exclude false positives. Following assembly and genome annotation, the pathogen carriage status of each species was determined (Figure 3). Through comprehensive analysis of the different types of pathogens carried by the flies and cockroaches, it was found that the flies and cockroaches exhibited some common intestinal bacteria, such as *Escherichia coli*, *Salmonella* spp., *Pseudomonas aeruginosa*, and others; however, both species still had most of the pathogens that were unique to them. For example, only *Salmonella typhi* and *Enterococcus faecium* were detected in flies. Only *Acinetobacter johnsonii* and *Aspergillus flavus* were detected in cockroaches. According to the analysis of different regions, compared with other regions, the distribution of pathogens in Southeast Asia was found to be more concentrated and extensive, with high-concentration pathogens accounting for 42% of the top 50 pathogens and more highly pathogenic pathogens that we usually pay close attention to, while the proportion of West Asia was the least, at only 14%.

## 4. Discussion

Flies and cockroaches are common vectors whose activities can pose a threat to public health, especially through mechanical or biological transmission of various pathogens [4]. In this study, imported flies and cockroaches brought in by ships, containers, aeroplanes, and other means of transportation were collected at 13 ports in Shenzhen, China, from October 2023 to April 2024. The distribution of their quantity, species, and places of origin was determined, and the pathogens they carried were detected and analysed using qPCR and metagenomic sequencing techniques.

Among the fly samples, the proportion of *M. domestica vicina* was found to be the highest, reaching 27.59%, possibly due to its high reproductive capacity and remarkable adaptability to various environments. Among the cockroach samples, *B. germanica* had the highest proportion at 66.47%, likely due to its widespread distribution and high reproductive capacity worldwide [13]. Further statistical analysis by place of origin revealed that the fly and cockroach samples intercepted at the ports mainly originated from continents such as Asia, Africa, and Europe. Shenzhen, an important hub for international exchange in China, has a subtropical monsoon climate that provides a favourable environment and conditions for the reproduction of flies and cockroaches. Therefore, monitoring and researching vectors and the pathogens they carry, which are imported through ports, can prevent the spread of infectious diseases via these vectors at border ports.

Given the strict quality requirements of metagenomic testing for sample detection [14], a fully automated nucleic acid and protein analysis system was used to screen the samples before library construction. Only samples that met the quality standards were included in the subsequent sequencing analysis. The electrophoresis results showed significant differences in the nucleic acid quality of the fly and cockroach samples. Specifically, the probability of nucleic acid degradation was higher in fly samples than in cockroach samples. Banerjee et al. reported that degradative enzymes in the intestines of flies could lead to easier nucleic acid degradation, inhibiting the extraction results [15].

An in-depth analysis of the pathogens carried by different species from different geographical regions revealed that flies carried *E. coli*, *P. aeruginosa*, and *S. aureus* from all four regions (Southeast Asia, East Asia, South Asia, and West Asia), whereas cockroaches carried *E. coli*, *E. faecalis*, and *S. aureus*. Notably, unique pathogens were detected in flies and cockroaches from each region: *Campylobacter jejuni* was detected in flies from Southeast Asia, *B. anthracis* from East Asia, *Bacteroides fragilis* from South Asia, and *B. bronchiseptica* from West Asia. In cockroaches, *B. fragilis* was detected in Southeast Asia, *Clostridium tetani* in East Asia, and *B. cereus* in West Asia. These pathogens pose serious threats to human health, causing symptoms such as diarrhoea, muscle spasms, and respiratory distress [16,17,18,19]. Through comprehensive analysis, the number of imported vectors from Southeast Asia was found to be the largest, and the pathogens carried were widely distributed and high in concentration. We speculate that Southeast Asia may be economically underdeveloped, with a poor sanitary and medical environment, and mostly desert areas, creating a good living environment for vectors and the pathogens they carry. Therefore, routine detection protocols and targeted and customised detection plans are essential to deal with vectors from different places of origin. This will facilitate complete screening and early warning tasks. Notably, the key pathogen *V. cholerae* was detected in flies from Southeast Asia and West Asia. A review of digestive disease news summaries from the corresponding period revealed that the cholera epidemic was spreading rapidly in places such as Singapore and Yemen, underscoring the effectiveness and early warning capability of detecting pathogens carried by imported vectors. Unfortunately, due to the small number of samples from South Asia, the conclusions may not be comprehensive.

According to statistics [20], from 2018 to 2022, most of the ports seized by vectors across the country are trade-developed areas, and Shenzhen, as an international port, has many import places and high frequency. Moreover, the risk of spreading new outbreaks of infectious diseases in the region is also greater, so more attention should be paid to it. In the past, the detection of pathogens carried by vectors was mostly performed by traditional detection methods, and multi-pathogen detection was relatively rare. In particular, the application of metagenomic sequencing to find pathogens is not a routine process at ports. Although many of the contents of this study need to be further verified and strengthened, it also suggests the importance of multi-pathogen detection.

As for the analysis of detection methods, the positive results for *E. coli*, *Salmonella*, *V. cholerae*, and *S. aureus* detected using qPCR were much lower than those detected using metagenomic sequencing. Although qPCR is the gold standard for pathogen detection [21,22,23], the use of this technique relies on known target sequences containing conserved primer targets. Therefore, the above preconditions are not always met. However, metagenomic sequencing may have higher sensitivity for certain pathogens [24,25], offering advantages such as unbiased detection, functional analysis potential, and a broader detection range [26,27,28]. In previous studies, researchers have systematically compared qPCR and metagenomic detection and concluded that although qPCR has absolute advantages in high-abundance gene detection, it still needs the coordination and supplement of metagenomic detection for genes with low abundance and high sensitivity and is susceptible to the influence of primer target mutations [29]. Therefore, in future microbial research and clinical diagnosis, more suitable methods need to be selected according to the actual situation, and metagenomic sequencing and qPCR can also be combined to obtain more accurate and comprehensive results.

This study had some limitations. First, the port may have been negligent in the processing and storage conditions of the samples, resulting in the degradation of pathogens carried by some samples and making the test results inaccurate. Second, the limited sample size and non-comprehensive selection of sample types may have restricted the information that could be obtained on the pathogens carried by vectors [30,31]. Again, due to the uncertainty of imported vectors captured by customs, limited or missing sample numbers from certain regions may have limited our overall analysis. Finally, qPCR and metagenomic detection have high requirements for the quality of nucleic acid samples, and the nucleic acid extraction process was not optimised in this study. In future work, we intend to standardise sample collection and preservation while expanding sample collection and analysis to include samples from more environments, supplementing the absence of pre-sample processing, and increasing focus on vector surveillance to better understand imported vectors and improve early warning systems.

## 5. Conclusions

In conclusion, we conducted detailed monitoring and analysis of pathogens carried by imported vectors intercepted by Shenzhen Customs at ports from 2023 to 2024. The findings of this study provide a basis for understanding the current status of pathogens at Shenzhen port and references for predicting future pathogen outbreak trends and formulating effective control strategies.

## Figures and Tables

**Table 1:** Test results of pathogens carried by imported flies.

Species	*Vibrio cholerae*	*Salmonella*	*Shigella*	*Escherichia coli* O157	*Staphylococcus aureus*
*Blattella germanica*	1/113 (0.88)	0/113 (0.00)	1/113 (0.88)	0/113 (0.00)	10/113 (8.85)
*Periplaneta australasiae*	0/4 (0.00)	0/4 (0.00)	0/4 (0.00)	0/4 (0.00)	0/4 (0.00)
*Periplaneta americana*	0/51 (0.00)	0/51 (0.00)	0/51 (0.00)	0/51 (0.00)	1/51 (1.96)
*Hebardina concinna*	0/2 (0.00)	0/2 (0.00)	0/2 (0.00)	0/2 (0.00)	0/2 (0.00)
Total	1/170 (0.59)	0/170 (0.00)	1/170 (0.59)	0/170 (0.00)	11/170 (6.47)

Number (number of positives/number of samples), with positive rate in parentheses (%).

**Table 2:** Test results of pathogens carried by imported cockroaches.

Species	*Vibrio cholerae*	*Salmonella*	*Shigella*	*Escherichia coli* O157	*Staphylococcus aureus*
*Parasarcophaga albiceps*	1/15 (6.67)	0/15 (0.00)	0/15 (0.00)	0/15 (0.00)	1/15 (6.67)
*Fannia leucosticta*	0/8 (0.00)	0/8 (0.00)	0/8 (0.00)	0/8 (0.00)	0/8 (0.00)
*Chrysomya megacephala*	0/35 (0.00)	0/35 (0.00)	0/35 (0.00)	0/35 (0.00)	2/35 (5.71)
*Anthomyia illocata*	0/8 (0.00)	0/8 (0.00)	0/8 (0.00)	0/8 (0.00)	0/8 (0.00)
*Musca domestica vicina*	0/96 (0.00)	0/96 (0.00)	0/96 (0.00)	2/96 (2.08)	4/96 (4.17)
*M. domestica*	0/82 (0.00)	0/82 (0.00)	0/82 (0.00)	2/96 (2.08)	6/96 (6.25)
*Sarcophagidae*	0/2 (0.00)	0/2 (0.00)	0/2 (0.00)	0/2 (0.00)	0/2 (0.00)
*Musca sorbens*	0/1 (0.00)	0/1 (0.00)	0/1 (0.00)	0/1 (0.00)	0/1 (0.00)
*Lucilia sericata*	0/6 (0.00)	0/6 (0.00)	0/6 (0.00)	0/6 (0.00)	0/6 (0.00)
*Lucilia cuprina*	0/10 (0.00)	0/10 (0.00)	0/10 (0.00)	0/10 (0.00)	1/10 (10.00)
*Musca pattoni*	0/34 (0.00)	0/34 (0.00)	0/34 (0.00)	0/34 (0.00)	1/34 (2.94)
*Fannia canicularis*	1/44 (2.27)	0/44 (0.00)	0/44 (0.00)	0/44 (0.00)	0/44 (0.00)
*Neomyia timorensis*	0/6 (0.00)	0/6 (0.00)	0/6 (0.00)	0/6 (0.00)	0/6 (0.00)
*Syrphidae*	0/1 (0.00)	0/1 (0.00)	0/1 (0.00)	0/1 (0.00)	0/1 (0.00)
Total	2/348 (0.57)	0/348 (0.00)	0/348 (0.00)	4/348 (1.15)	15/348 (4.31)

Number (number of positives/number of samples), with positive rate in parentheses (%).

## Data Availability

The data will be made available upon request.

## Supplementary Materials

The following supporting information can be downloaded at https://www.mdpi.com/article/10.3390/tropicalmed10020057/s1. Figure S1: The whole early warning evaluation process; Figure S2: Part of the quality control data result chart.

## Abbreviations

The following abbreviations were used in this manuscript:

VBD	Vector-borne disease;
qPCR	Polymerase chain reaction.
