# Supplementary material for: Monitoring of Pathogens Carried by Imported Flies and Cockroaches at Shenzhen Ports
Source: Trop Med Infect Dis. 2025 Feb 17;10(2):57. doi: 10.3390/tropicalmed10020057 (PMC11860353; doi:10.3390/tropicalmed10020057)

Figure S1. The whole early warning evaluation process.

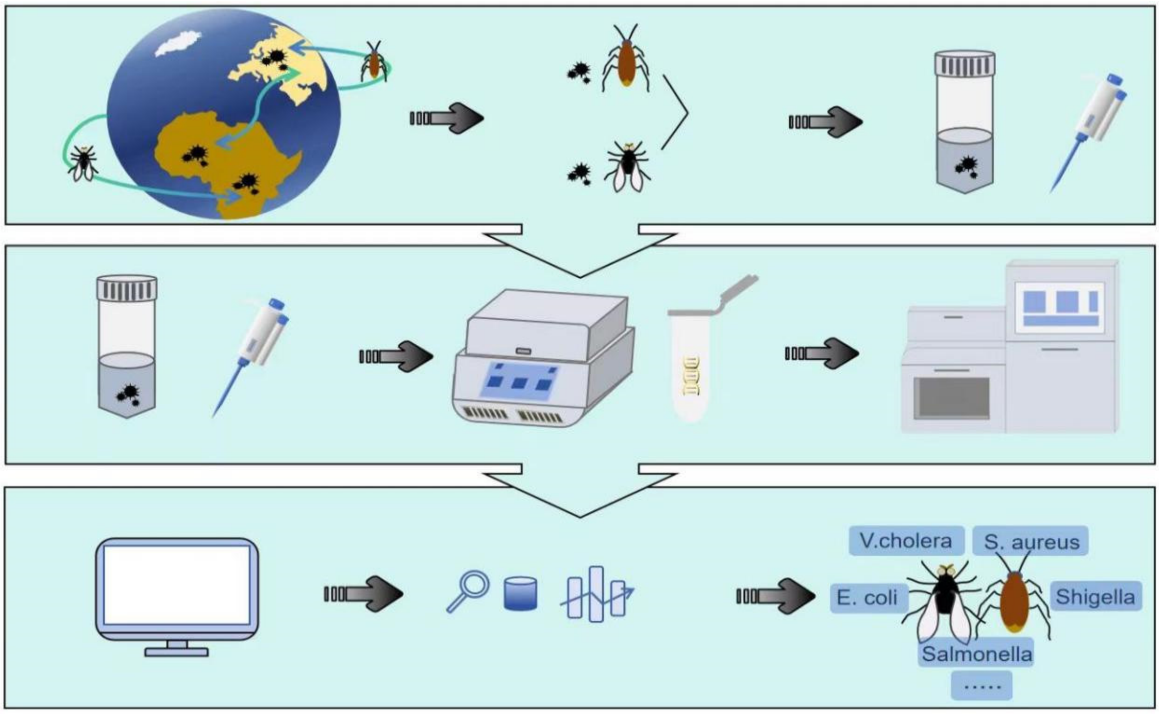

Figure S2. Part of the quality control data result chart.

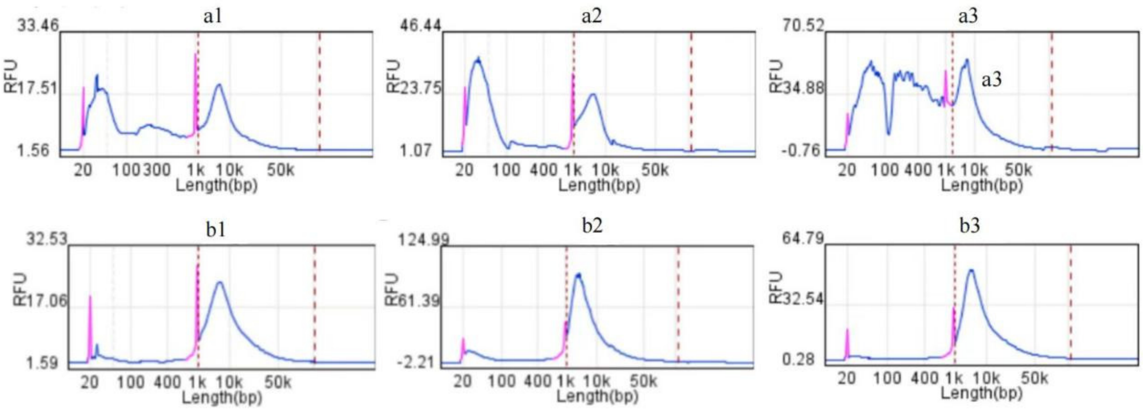

Supplement: Supplementary file 1 [file tropicalmed-10-00057-s001.zip › tropicalmed-3439261-supplementary.pdf]
